# Exogenous xylose trapped threonine to regenerate Amadori compounds and accelerated pH decline impeding pyrazines formation upon thermal treatment of Amadori compounds revealed by sotope labeling

**DOI:** 10.1016/j.crfs.2026.101433

**Published:** 2026-05-06

**Authors:** Pusen Chen, Baishun Hu, Zuman Dou, Fei Meng, Qiong Deng

**Affiliations:** a Department of Food and Environmental Engineering, Chuzhou Vocational and Technical College, Chuzhou, 239000, China; b Enshi Tujia and Miao Autonomous Prefecture Academy of Agricultural Sciences, Enshi, 445000, China; c College of Ocean Food and Biological Engineering, Jimei University, Xiamen, 361021, China

**Keywords:** Isotope labeling method, Amadori rearrangement products, Inhibition effect, Pyrazine compounds

## Abstract

The isotope labeling method was adopted to reveal the mechanism by which exogenous xylose inhibited pyrazine compounds generation during thermal processing of Thr-ARP in this study. The [^13^C^5^]-D-xylose/Thr-ARP model reaction system was heated, the reaction products under 120 °C and an initial pH 7.5 were tracked and determined by UPLC-MS, the ion fragmentation mechanism indicated [^13^C^5^]-Thr-ARP was formed. It was proposed [^13^C^5^]-D-xylose trapped threonine regenerated from Thr-ARP and then underwent Amadori rearrangement to form [^13^C^5^]-Thr-ARP. Thr-ARP's regeneration delayed the release of regenerated threonine and partially hindered Strecker degradation to generate pyrazine compounds. In addition, the self-cleavage of exogenous xylose caused an increase in the concentrations of 1-deoxyxylosone and 3-deoxyxylosone. The organic acids generated from the cleavage of deoxyxylosones led to an accelerated decrease in pH, reducing regenerated threonine's nucleophilicity. The synergistic effect of the above two factors led to a reduction in the concentration and variety of pyrazine compounds produced by the thermal degradation of Thr-ARP.

## Highlights

• Xylose inhibited pyrazines formation during Amadori compounds (ARP) degradation.
• Isotope labeling revealed that [^13^C^5^]-xylose and threonine formed [^13^C^5^]-ARP.
• Xylose accelerated pH decline rate during the ARP degradation.
• ARP's reforming and pH accelerated decrease jointly inhibited pyrazines formation.

## Graphical abstract

**Figure:**
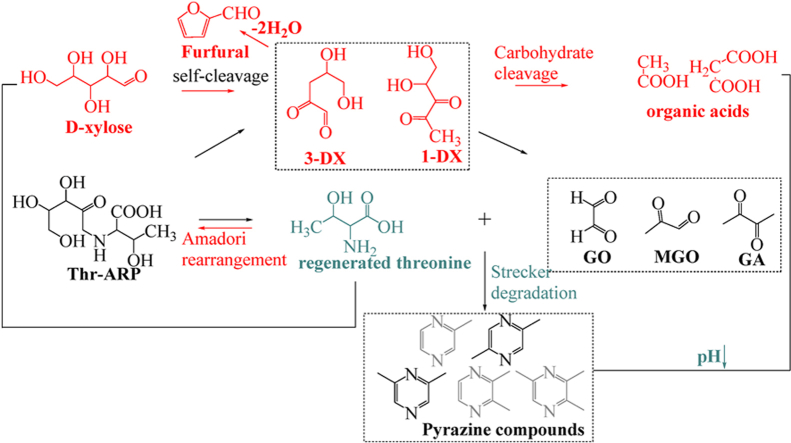

## 1 Introduction

The appealing color and flavor profile of many heated foods arises primarily from the Maillard reaction (Davidek et al., 2003). The reaction mechanism involves an advanced cascade of chemical steps initiated by the interaction between reducing sugars' carbonyl groups and amino groups found in amino acids, peptides, or similar nitrogen-containing compounds (Muir, 2007). Amadori compounds, which form during the initial stage of the Maillard process, act as essential non-volatile intermediates that precede the generation of further reaction products (Cui et al., 2021). Over recent years, they have been extensively documented in both raw materials and thermally processed food items (Molero et al., 1997). Compared to later-stage Maillard derivatives, Amadori compounds exhibit greater stability yet remain crucial reactive species that catalyze the development of varied advanced glycation end-products, thereby generating the distinctive flavors, odors, and hues associated with heated foods (Troise et al., 2016). Due to their properties, these compounds are viewed as promising candidates for replacing traditional Maillard-derived flavorings in food industry applications.

A number of studies have demonstrated that the thermal processing of Amadori rearrangement products generates a diverse spectrum of volatile flavor compounds. Importantly, the quantity and rate of formation of these volatiles markedly exceed those produced by simple binary mixtures of reducing sugars and amino acids subjected to equivalent heating conditions (Cui et al., 2019). Evidence indicated that the spectrum of flavor compounds generated from ARPs was narrower than that derived from binary reducing sugar/amino acid models (Feng et al., 2022). Notably, pyrazine compounds—key compounds within the Maillard reaction product spectrum—were absent from the volatile profile generated by the thermal decomposition of ARPs (Zhou et al., 2022). Pyrazines contribute significantly to the sensory appeal of numerous thermally prepared foods, primarily through imparting the quintessential roasted, nutty, earthy, cereal-like, and savory meat notes associated with their flavor profiles (Yu et al., 2021). These distinctive organoleptic characteristics enable pyrazines to significantly shape a food's sensory profile, establishing them as indispensable ingredients within the flavor and food technology sectors (Chen et al., 2023). The primary route leading to the generation of pyrazines has widely been ascribed to the Strecker degradation mechanism, initiated by the reaction between α-dicarbonyl compounds and available amino acids (Deng et al., 2022). As a result, Strecker aldehydes and α-aminocarbonyl derivatives were produced through this reaction. Subsequently, further condensation reactions between two α-aminocarbonyl compounds could yield dihydropyrazine, which, upon oxidation, results in the corresponding pyrazines (Liu et al., 2022). Deng's study involved a comparative examination of pyrazine and precursor dynamics during the thermal decomposition of both a methionine-glucose binary system and the corresponding Amadori compounds derived from methionine and glucose (Deng et al., 2022). The investigation concluded that concurrent, adequate concentrations of α-dicarbonyls and amino acids, coupled with a reduction in pH, were pivotal in governing pyrazine synthesis (Deng et al., 2022).

Studies have shown that the production of pyrazines in thermally processed Amadori rearrangement product systems can be enhanced through several means: increasing alkalinity, applying higher temperatures, incorporating additional amino acids or peptides, and prolonging heating time (Xia et al., 2022). The addition of external glutamic acid during the thermal breakdown of glutamic acid-galactose Amadori rearrangement products significantly elevated pyrazines yields (Pan et al., 2022). An analogous effect was also noted in the glutathione-ribose ARP system (Feng et al., 2023), and likewise for the ARP generated from glucose and L-alanyl-L-glutamine (Xia et al., 2022). The process through which supplementary amino acids and peptides enhance pyrazine yield in the ARP model was also explored. Utilizing an isotope labeling method, Chen et al. identified that exogenous threonine plays two primary roles in promoting the generation of pyrazines during thermal decomposition process of the ARP system: (1) It facilitated the conversion of threonine-derived Amadori rearrangement products into their corresponding free threonine, deoxyxylosones, and low-molecular-weight dicarbonyl species, thus securing an adequate precursor pool for Strecker degradation. (2) It functioned as a pH buffer, increasing the availability of unprotonated amino groups and establishing more conducive ambient conditions for Strecker degradation to proceed (Chen et al., 2023).

Along with amino acids, reducing sugars constituted a principal group of reactants in the Maillard process, where they decomposed to yield α-dicarbonyl compounds (Scalone et al., 2015), which serve as crucial precursors for pyrazine formation. Consequently, it is theoretically plausible that the incorporation of reducing sugars into the Maillard reaction system could substantially enhance the generation of pyrazines during the thermal degradation process. Contrary to the initial conjecture, Zhang et al. discovered that in the model of mono-glycated Lys-ARP, the addition of xylose significantly increased the yield of furans while delaying lysine release. This delay subsequently inhibited the early formation of pyrazines at 100 °C (Zhang et al., 2025). The potential inhibitory impact of exogenous xylose on the thermal decomposition of diverse amino acid-derived ARPs and the consequent formation of pyrazines remains to be fully elucidated. Moreover, the precise pathways through which exogenous xylose impeded the liberation of regenerated amino acids and thereby attenuated ARP thermal degradation and subsequent pyrazines formation via alternative reaction routes have not been clarified. The objective of this paper is to investigate whether exogenous xylose can promote/inhibit pyrazine compounds formation during the thermal treatment of Thr-ARP, and to elucidate the mechanisms by which exogenous xylose exerted a promoting/inhibiting effect on the thermal degradation of Thr-ARP to produce pyrazine compounds. Two model reaction systems, Thr-ARP and Thr-ARP/xylose, were established. Throughout the reaction processes, the variations in key precursors (such as Amadori compounds, threonine, deoxyxylosones, glyoxal, methylglyoxal, and diacetyl) as well as critical reaction parameters, including pH, which were implicated in the formation of pyrazine compounds, were closely monitored. Isotopically labeled xylose was used to trace the fate of xylose during the ARP degradation reaction. Based on this, a reaction pathway was proposed to elucidate the involvement of exogenous xylose in ARP degradation, consequently yielding mechanistic insight into how exogenous xylose regulated pyrazine generation in the course of Thr-ARP thermal degradation.

## 2 Materials and methods

### 2.1 Raw materials and chemicals

[^13^C^5^]-D-xylose (99%), trimethylpyrazine, along with 2,3-dimethylpyrazine, 2,5-dimethylpyrazine, 2,6-dimethylpyrazine, and methylpyrazine, were sourced from Sigma-Aldrich Chemical Co. (Shanghai, China). L-Threonine, D-xylose, glyoxal (40% aqueous solution), methylglyoxal (40% aqueous solution), diethylenetriaminepentaacetic acid, o-phenylenediamine, ammonium hydroxide, and ammonium hydroxide and 4-(2-hydroxyethyl)-1-piperazineethanesulfonic acid (HEPES) were acquired from Macklin Biochemical Co. (Shanghai, China). Furfural (99% purity), formic acid, methyl alcohol and acetonitrile of high performance liquid chromatography (HPLC) grade were obtained from Sinopharm Chemical Reagent Co. Ltd. (Shanghai, China), Thr-ARP and 3-deoxyxylosone-quinoxaline (3-DX-quinoxaline) were synthesized within our laboratory.

### 2.2 Preparation, purification, and identification of Thr-ARP

The procedures used for synthesizing, purifying, and characterizing Thr-ARP were adapted from an earlier study by Chen and colleagues, with certain adjustments implemented (Chen et al., 2022). Initially, D-xylose and L-threonine were mixed and then brought into solution using ultrapure water, yielding final concentrations of 0.5 mol/L for D-xylose and 0.25 mol/L for L-threonine. Subsequently, the pH was precisely adjusted to 7.50 (±0.02). Subsequently, a 50 mL portion of the mixture was incubated for 80 min in a temperature-controlled water bath set at 90 °C. Following this, the pH was readjusted to 7.50 (±0.02) by adding a 6 mol/L sodium hydroxide solution through titration. The sample then underwent vacuum rotary evaporation at 95 °C for 12 min to obtain Thr-ARP, which was reconstituted in 20 mL of ultrapure water. The purification of Thr-ARP from the reconstituted solution followed the methodology described by Chen and colleagues (Chen et al., 2022). For preliminary purification, Thr-ARP was processed using a column filled with 200-400 mesh Dowex 50WX4 ion-exchange resin (H^+^) sourced from suppliers including Acros Organics, SERVA, and J&K (Beijing, China). Subsequently, a semipreparative RP-HPLC column (C_18_) was employed for further purification. The purified Thr-ARP was characterized via ^1^H NMR spectroscopy on a Bruker DRX 400 MHz instrument and by UPLC-ESI-MS analysis using a Waters Synapt MALDI Q-TOF mass spectrometer.

### 2.3 Model reaction of Thr-ARP and D-xylose

Different amounts of D-xylose were incorporated into the 20 mmol/L Thr-ARP solutions, ensuring that the final concentrations of D-xylose in these solutions were 0, 10, 20, 30, and 40 mmol/L, respectively. All previously mentioned solutions were brought to a pH of 7.5 (±0.02) by adding a 5 mmol/L sodium hydroxide solution, and subsequently transferred into pressure-resistant glass bottles. All glass bottles containing the sample solutions were then placed in an oil bath maintained at a temperature of 120 °C. After undergoing heat treatment for varying durations (0, 30, 60, 90, 120, and 150 min), all samples were promptly placed in an ice bath to facilitate cooling before being removed for subsequent testing (Chen et al., 2022).

### 2.4 Quantitative analysis of Thr-ARP and L-Threonine (Thr)

Quantification of both threonine (Thr) and Thr-ARP was performed using HPLC equipped with an evaporative light-scattering detector (ELSD). The separation was carried out on an Xbridge Amide column (4.6 × 150 mm, 5 μm; manufactured by Waters Corporation, Milford, MA, U.S.A.). The mobile phase comprised two components: component A contained 0.1% formic acid in ultrapure water, while component B consisted of 0.1% formic acid in acetonitrile. An 8 μL sample aliquot was injected for each run. The gradient elution program was implemented with the following profile: from 0 to 3 min, mobile phase A was maintained at 30%; between 3 and 12 min, it increased from 30% to 35%; from 12 to 15 min, it further rose to 42%; between 15 and 17 min, it decreased back to 35%; and finally, from 17 to 20 min, it was set at 72%. Employing a consistent flow rate of 0.8 mL/min yielded retention times of 12.8 min for Thr-ARP and 10.6 min for threonine. The calibration equation of Thr-ARP was: *y* = 3.429 × 10^6^*x*−2.548 × 10^4^, with a coefficient of determination (R^2^) of 0.998, whereas the derived detection limit (LOD) and quantitation limit (LOQ) values were determined to be 0.199 μmol/L and 0.601 μmol/L, respectively. The calibration curve for threonine was established as: *y* = 1.109 × 10^6^*x* − 5.23 × 10^5^, showing a correlation coefficient (R^2^) of 0.998 and providing detection and quantitation limits of 0.421 μmol/L and 1.223 μmol/L, respectively, *x* corresponded to concentration values, while *y* indicated the associated peak area measurements (Chen et al., 2024).

### 2.5 Quantitation of α-dicarbonyl compounds

The samples were analyzed for their α-dicarbonyl compound content via HPLC with a diode array detector (DAD; Waters 1525 system, Waters 2996 detector), following a previously established method (Yu et al., 2019). At the outset, the samples underwent derivatization. The OPD (0.5 wt%), HEPES (1 mol/L), and DTPA (12 mmol/L) were dissolved in ultrapure water to function as derivatizing agents. A 0.4 mL aliquot of the sample was mixed with an equal volume of the derivatization reagent, followed by sealing the vial and incubating it in darkness at 30 °C for 4 h. Separation for analysis was conducted on a Waters SunFire C_18_ column, measuring 150 mm × 4.6 mm and packed with 5 μm particles. Detection was performed at 315 nm with a 5 μL sample injection volume. The mobile phase comprised two components: (A) ultrapure water containing 0.1% formic acid and (B) methanol with 0.1% formic acid. Chromatographic separation was performed at a column temperature of 30 °C and a constant flow of 0.6 mL/min, according to the following gradient: from 0 to 4 min, 35% B; between 4 and 12 min, increasing to 65% B; from 12 to 15 min, returning to 30% B; and holding at 30% B from 15 to 17 min. The column oven was set at 35 °C throughout the analysis. The calibration curve, limit of detection (LOD), and limit of quantification (LOQ) results were presented below: 3-DX (*y* = 4.79 × 10^6^*x* − 2.19 × 10^4^, LOD = 0.59 μmol/L, LOQ = 2.03 μmol/L), 1-DX (*y* = 4.78 × 10^6^*x* − 3.22 × 10^4^, LOD = 0.58 μmol/L, LOQ = 2.29 μmol/L). GO (*y* = 2.47 × 10^3^*x* − 7.83 × 10^4^, LOD = 0.57 μmol/L, LOQ = 2.32 μmol/L). MGO (*y* = 3.46 × 10^3^*x* − 1.44 × 10^4^, LOD = 0.47 μmol/L, LOQ = 2.08 μmol/L). Here, *x* denoted the peak area, and *y* corresponded to the analyte concentration in mmol/L.

### 2.6 Quantitation of furfural

HPLC equipped with a UV detector was employed to quantify the furfural (Pham et al., 2020). Furfural was monitored at a wavelength of 277 nm using a SunFire C_18_ column (Waters; 150 mm × 4.6 mm, 5 μm) maintained at 30 °C. The eluent consisted of ultra-pure water (solvent A) and methanol (solvent B), delivered at a flow rate of 0.8 mL/min. The initial gradient conditions were set at 85% A, held from 0 to 15 min. The regression model for furfural quantification was expressed as: *y* = 9.01 × 10^−8^ *x* − 5.81 × 10^−2^, (R^2^ = 0.996, RT = 7.6 min, LOQ = 1.438 μmol/L, LOD = 0.531 μmol/L), where *x* represented the peak area, *y* represented the concentration (mmol/L). Fig. S1 illustrated the yield of furfural produced during the thermal interaction of Thr-ARP with different concentrations of D-xylose.

### 2.7 Analysis and measurement of pyrazines

A 3 mL portion of the sample, which included 2 μL of a 1,2-dichlorobenzene in methanol solution (0.018 μg/μL), was transferred into a 15 mL vial and sealed with a PTFE/BUTYL septum. Volatile flavor compounds produced by heating were collected using a manual headspace solid-phase microextraction (HS-SPME) system equipped with a divinylbenzene/Carboxen/polydimethylsiloxane fiber (Supelco, Bellefonte, PA, USA). While maintained at 55 °C and agitated with a magnetic stirrer for 35 min, the samples underwent headspace extraction with the fiber exposed. The fiber was then introduced into the gas chromatograph injector, maintained at 250 °C, for 8 min of desorption in splitless mode. The extracted volatiles were chromatographically separated using a DB-Wax capillary column (J&W Scientific, Folsom, CA, USA; 30 m × 0.25 mm × 0.25 μm). The column temperature was controlled using the following sequence: starting with an isothermal hold at 40 °C for 3 min, then increasing at 8 °C/min to 80 °C and holding for 3 min, followed by a ramp at 5 °C/min to 150 °C with a 2-min hold, and finally rising at 7 °C/min to 250 °C for a final 2-min isothermal period. A steady helium carrier gas flow was maintained at 1.5 mL/min. The mass spectrometer functioned with an ion source temperature set to 230 °C and employed electron impact ionization at 70 eV, scanning a mass-to-charge range of 30 to 500. Compound identification was achieved by matching both the mass spectra and experimentally determined retention indices (relative to n-alkanes C7-C30) with entries in the NIST 17 mass spectral database. For external calibration, a consistent amount of internal standard (2 μL of 1,2-dichlorobenzene) was introduced into standard solutions at different concentrations. Calibration plots were subsequently generated by graphing the relationship between the analyte-to-internal-standard peak area ratio and concentration. The calibration curves of pyrazines were shown below: methylpyrazine (*y* = 0.0426 *x* – 0.0009, R^2^ = 0.997, RT = 13.989 min); 2,5-dimethylpyrazine (*y* = 0.0512 *x*+ 0.0002, R^2^ = 0.9956, RT = 15.081); 2,6-dimethylpyrazine (*y* = 0.0609 *x*+ 0.0007, R^2^ = 0.9971, RT = 15.198); 2,3-dimethylpyrazine (*y* = 0.0183 *x*+ 0.0003, R^2^ = 0.9968, RT = 16.306); trimethylpyrazine (*y* = 0.0249 *x*+ 0.0004, R^2^ = 0.9982, RT = 17.268). In the calibration model, *y* denoted the ratio of the volatile standard's peak area to that of the internal standard, whereas *x* signified the corresponding ratio of their concentrations (Zhou et al., 2022).

### 2.8 Detection of ^13^C^5^-Thr-ARP by UPLC-MS/MS

The isotopic tracer ^13^C^5^-Thr-ARP was prepared by heating a mixture of ^13^C^5^-D-xylose (20 mmol/L) and Thr-ARP (20 mmol/L) in an oil bath at 120 °C for 20 min, with the reaction pH initially adjusted to 7.5. The resulting mixtures were analyzed directly by UPLC-MS/MS, employing electrospray ionization in negative mode (ESI^−^). Instrument settings included a collision energy of 5.0 eV, a capillary voltage of 3.5 kV, cone and extraction cone voltages set at 20 V and 4.0 V respectively. The source and desolvation temperatures were maintained at 100 °C and 400 °C, while cone gas and desolvation gas flows were 50 L/h and 700 L/h. Full-scan spectra from m/z 50 to 1000 were recorded using a scan time of 0.5 s and an interscan delay of 0.02 s. For the LC-MS separation, an ultra-performance liquid chromatography system equipped with a CSH C_18_ column (2.1 mm × 100 mm, 1.7 μm; Waters, USA) was used at 45.0 °C. The flow rate was set to 0.3 mL/min with a 5 μL injection volume. The mobile phases were 0.1% formic acid in water (A) and pure acetonitrile (B). The gradient program was: 2% A initially; increased linearly to 20% A at 5 min, 40% A at 8 min, and 80% A at 10 min; followed by re-equilibration to 2% A by 12 min. All data were collected and processed using MassLynx software (v4.1; Milford, MA, USA).

### 2.9 pH measurement

The pH values of all samples were determined by placing the solution in an ice bath to cool it to approximately 25 °C (±0.2 °C) following the designated reaction times of 0, 30, 60, 90, 120, and 150 min.

### 2.10 Statistical analysis

Data were reported as mean ± standard deviation, with all experimental treatments conducted in three independent replicates. Statistical analysis was performed using SPSS software (version 19.0; IBM, Armonk, NY), and differences were considered significant at *p* < 0.05.

## 3 Results and discussion

### 3.1 Inhibition effect of exogenous xylose on the pyrazines formation through Thr-ARP thermal degradation

To examine how xylose addition affected pyrazine compounds production during the thermal degradation of threonine-derived Amadori rearrangement products (Thr-ARP), mixtures with 20 mmol/L Thr-ARP were prepared alongside varying concentrations of D-xylose (0–40 mmol/L) and thermally processed for 150 min. The yield of pyrazine compounds in each sample after the reaction was quantitatively detected by GC-MS. Refer to Table 1 for details, under the specified conditions of pH 7.5 and 120 °C, Thr-ARP degradation itself generated numerous pyrazine compounds in significant amounts. In our previous research, it was found that Thr-ARP alone could not effectively generate pyrazine compounds under the conditions of pH 7 and 120 °C (Chen et al., 2023). Therefore, a higher initial pH could promote the thermal degradation of Thr-ARP to generate pyrazine compounds, which is similar to Xia's research (Xia et al., 2022). The variety and levels of pyrazine compounds produced by L-Alanyl-L-glutamine and glucose-derived ARP, as well as those generated under similar conditions at pH 7.5, were found to be significantly higher than the levels of pyrazines formed at pH 7 under identical experimental conditions. Thr-ARP were rapidly cleaved into deoxyxylosones and regenerate threonine upon heating. Deoxyxylosones broke down through retro-aldol pathways or oxidative cleavage, resulting in α-dicarbonyls like GO and MGO. Resulting α-dicarbonyls then engaged in Strecker degradation with reformed amino acids to generate α-aminoketones. Subsequently, these α-amino ketones underwent carbonyl-amine condensation in pairs, facilitating the removal of two molecules of water and generating dihydropyrazines. Finally, dihydropyrazines were oxidized to yield pyrazine compounds (Low et al., 2007). Therefore, the phenomenon described above, in which a higher pH facilitated the generation of pyrazines, could be attributed to the enhanced nucleophilicity of regenerated threonine at elevated pH levels. This enhanced nucleophilic character facilitated more efficient interactions between reformed threonine and α-dicarbonyl species, culminating in pyrazine formation (Zhan et al., 2022).

**Table 1:** Concentration of pyrazine compounds formed by thermal reaction of Thr-ARP with different concentrations of D-xylose (The initial concentration of Thr-ARP was 20 mmol/L, the reaction temperature was 120 °C and heated for 150 min, the initial pH was 7.5).

		Concentration of pyrazine compounds produced by different model reactions			
Pyrazine compounds (μg/L)	ARP	ARP/Xyl (10 mmol/L Xyl)	ARP/Xyl (20 mmol/L Xyl)	ARP/Xyl (30 mmol/L Xyl)	ARP/Xyl (40 mmol/L Xyl)
Methylpyrazine	481.9710	288.5394	37.6816	9.7312	ND
2,5-Dimethylpyrazine	651.8778	457.1715	314.4900	264.1123	185.8277
2,6-Dimethylpyrazine	323.4797	240.2604	207.3104	178.3212	167.6570
2,3-Dimethylpyrazine	226.0624	136.8467	28.9632	4.6243	ND
Trimethylpyrazine	286.8923	206.0699	97.9533	34.5614	ND

On the other hand, and more importantly, data in Table 1 indicated an inverse relationship between xylose concentration and pyrazine compounds’ yield from Thr-ARP thermal degradation. Notably, when the amount of xylose added was 40 mmol/L, methylpyrazine, trimethylpyrazine, and tetramethylpyrazine were undetectable. Based on these findings, introducing supplemental D-xylose markedly suppressed pyrazine compounds generation during the thermal breakdown of Thr-ARP. This intervention not only lowered the concentration of pyrazine compounds but also diminished the variety of pyrazine compounds present. This phenomenon was highly atypical because the degradation of exogenous xylose could promote the production of α-dicarbonyl compounds, the precursors necessary for Strecker degradation. According to the known reaction pathway, xylose inclusion would be expected to promote pyrazine formation from Thr-ARP degradation. To elucidate the inhibitory role of additional xylose on pyrazine compounds synthesis during Thr-ARP thermal decomposition, we closely monitored the key precursors of pyrazine compounds as well as the degradation products of xylose throughout the entire degradation process of Thr-ARP.

### 3.2 Stimulatory effect of exogenous xylose on the generation of α-dicarbonyl compounds and its inhibitory effect on regenerated threonine

To elucidate the suppression mechanism of external xylose on pyrazine generation from heated Thr-ARP, key Strecker degradation precursors were measured across samples with different supplemental xylose levels. These reactants included regenerated threonine, α-dicarbonyl compounds. The upstream and downstream products of the above-mentioned reactants were also quantitatively detected. Based on these findings, it could be determined whether the insufficient availability of pyrazine precursors was responsible for the suppression of pyrazine compounds formation.

**Fig. 1:**
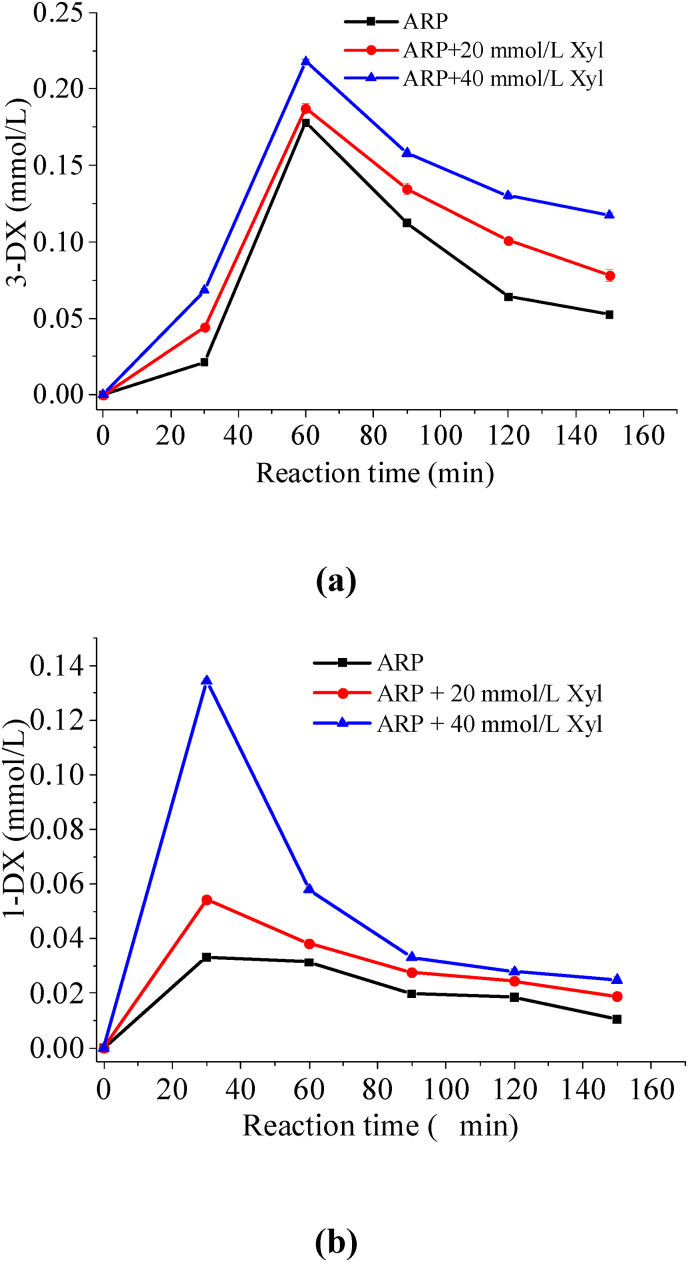
Time course of 3-DX (a) and 1-DX (b) concentration in Thr-ARP model and Thr-ARP/Xyl models during thermal reaction.

**Fig. 2:**
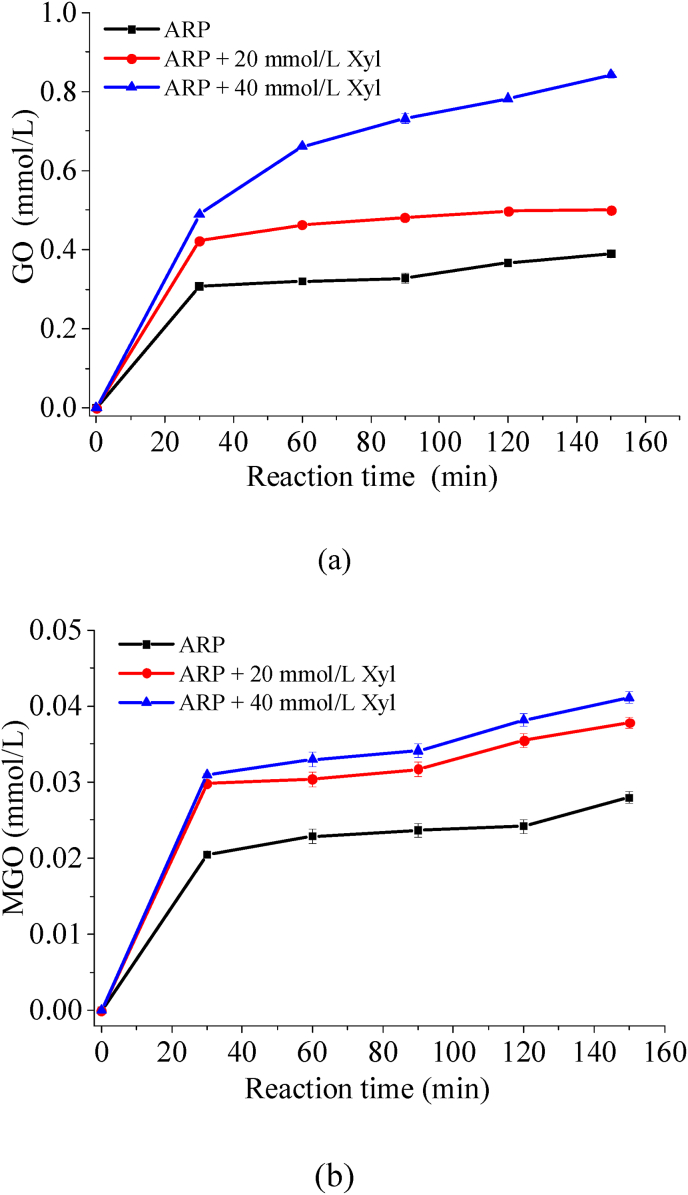
Time course of GO (a) and MGO (b) concentration in Thr-ARP model and Thr-ARP/Xyl models during thermal reaction.

As previously mentioned, regenerated threonine and α-dicarbonyl compounds could be considered direct precursor reactants in the synthesis of pyrazine compounds (Low et al., 2007). Moreover, the concentrations of the reaction precursors 1-DX and 3-DX, which generated short-chain α-dicarbonyls, were similarly tracked throughout the heating period. As illustrated in Fig. 1, the concentrations of both 3-DX and 1-DX exhibited a trend characterized by an initial increase followed by a subsequent decrease as heating progressed. The concentration of 3-DX peaked at 60 min of heating, whereas the concentration of 1-DX reached its peak at 30 min of heating. In contrast, the concentration of 1-DX was significantly lower than that of 3-DX. This result is consistent with earlier published data (Zhai et al., 2021). The underlying reason for this phenomenon was that the structure of 1-DX exhibited greater instability and a higher susceptibility to cracking, resulting in the production of low-molecular-weight α-dicarbonyl species (Thornalley et al., 1999). More notably, samples with xylose supplementation exhibited elevated levels of both 1-DX and 3-DX compared to those containing only Thr-ARP. There might be two reasons for this phenomenon: firstly, the addition of xylose facilitated the cleavage of Thr-ARP, thereby accelerating the formation of 1-DX and 3-DX. This observation was analogous to a previously reported scenario in which the introduction of exogenous threonine enhanced the fragmentation of Thr-ARP (Chen et al., 2023). Secondly, xylose itself underwent carbohydrate cleavage, resulting in the production of both 3-DX and 1-DX (Zhang et al., 2012). As illustrated in Fig. 2(a), the concentrations of GO and MGO also adhere to the aforementioned rule, exhibiting higher levels in samples with added exogenous xylose. Furthermore, this phenomenon became increasingly pronounced with the rising concentration of xylose added. This observation could be readily understood, as deoxyxylosones served as a precursor for both GO and MGO. Consequently, an increase in the concentration of this precursor would correspondingly lead to elevated levels of the products GO and MGO.

**Fig. 3:**
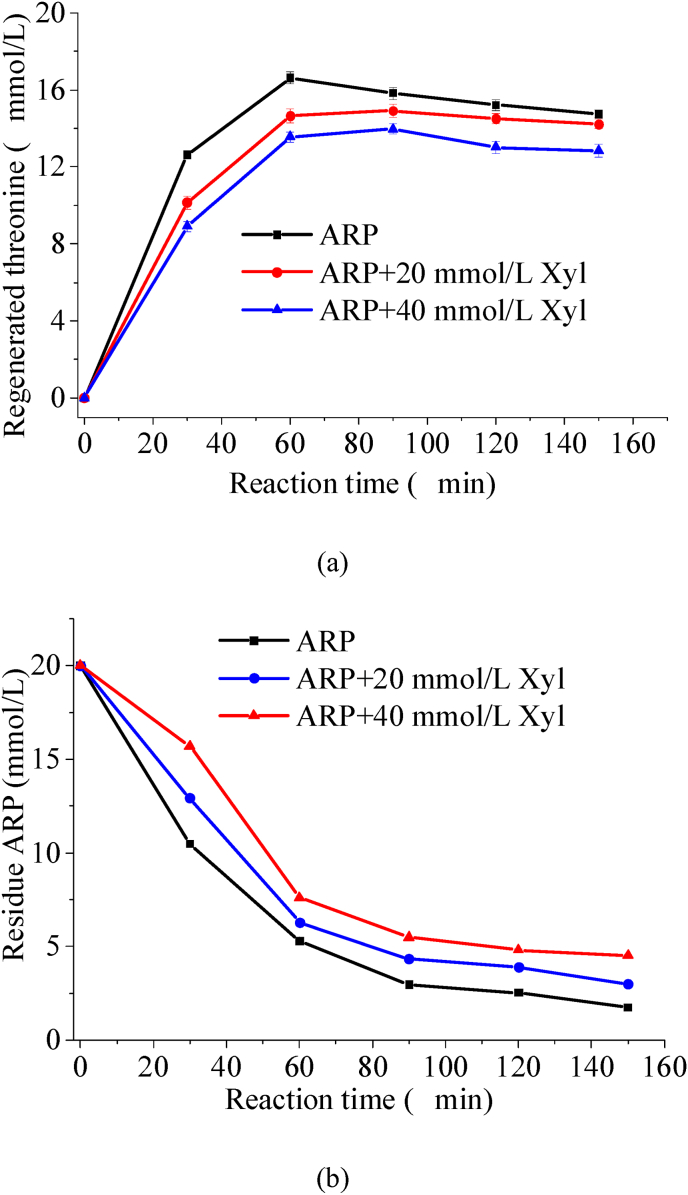
Time course of regenerated threonine concentration (a) and residue ARP concentration (b) in Thr-ARP model and Thr-ARP/Xyl models during thermal reaction.

To clarify which of the two aforementioned pathways was influenced by exogenous xylose in promoting the formation of 1-DX and 3-DX, we analyzed another product resulting from Thr-ARP cleavage: regenerated threonine. The results were presented in Fig. 3(a).

As the thermal reaction time was extended, the regenerated threonine was gradually released through Thr-ARP, leading to a progressive increase in its concentration. However, systems containing additional xylose demonstrated a marked reduction in reformed threonine levels. Furthermore, an increase in the amount of xylose added corresponded to a further decrease in the concentration of regenerated threonine. This finding was in stark contrast to the previous result, which indicated that the addition of threonine promoted an increase in regenerated threonine concentration (Chen et al., 2023). The results indicated that exogenous xylose did not facilitate the cleavage of Thr-ARP. Conversely, it was highly likely that exogenous xylose inhibited the cleavage of Thr-ARP. This observation explained why the concentration of regenerated threonine in the Thr-ARP samples with added exogenous xylose was lower than that in the samples without such addition. As illustrated in Fig. 3(b), the decomposition of Thr-ARP gradually increased with extended heating time, leading to a corresponding decrease in its concentration. In contrast, for samples of Thr-ARP that had exogenous xylose added, the rate of Thr-ARP decomposition was notably slower. Consequently, the residual concentration of Thr-ARP in the solution remained higher. Furthermore, as more exogenous xylose was introduced, the decomposition rate of Thr-ARP decreased further, resulting in an increased amount of Thr-ARP retained within the solution. This phenomenon clearly indicated that exogenous xylose delayed the cleavage of Thr-ARP, consequently reducing the rate of regenerated threonine production. The observed increases in the concentrations of GO, MGO, deoxyxylosones, furfural, and other compounds were primarily attributed to the carbohydrate cleavage of xylose itself. Due to the low concentration of regenerated threonine and the absence of the key precursor threonine, Strecker degradation could not proceed. This limitation resulted in an inability to effectively synthesize pyrazine compounds when exogenous xylose was added. However, additional research was required to elucidate the reaction pathways by which exogenous xylose modulated the degradation rate of Thr-ARP.

### 3.3 Reformation of Thr-ARP from exogenous xylose and regenerated threonine inhibiting pyrazines production revealed by isotope labeling

**Fig. 4:**
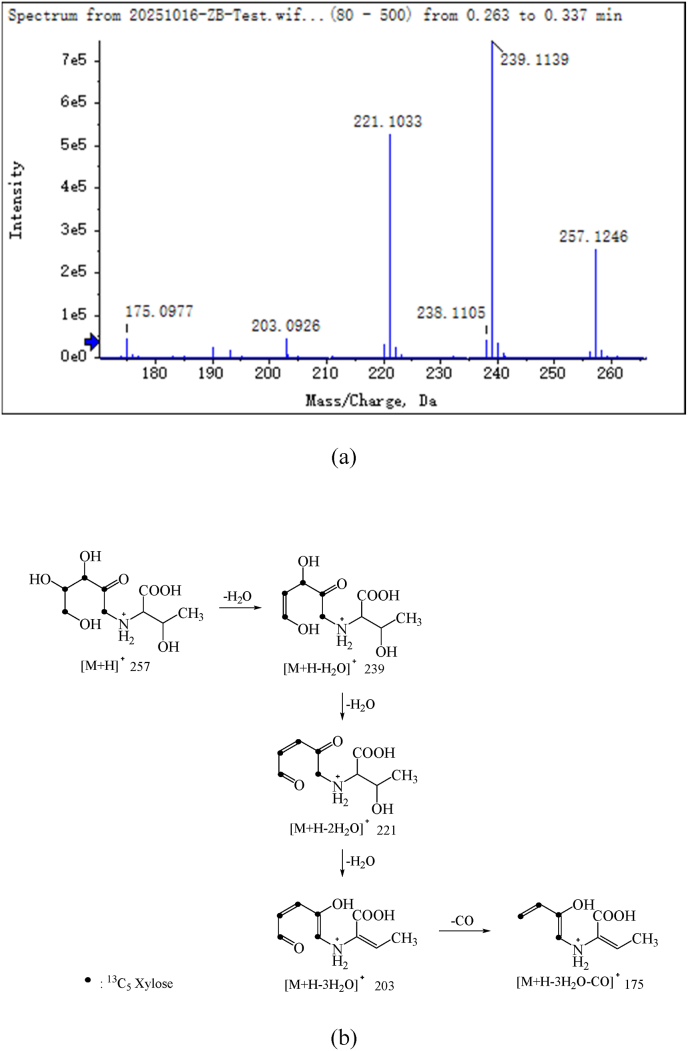
Total ion chromatogram (a) and MS/MS spectrum (b) of Thr-ARP formed by exogenous ^13^C^5^-xylose and regenerated threonine.

The results presented above elucidate the reason why the introduction of exogenous xylose prolonged the degradation of Thr-ARP, consequently leading to a reduced rate of regenerated threonine production. According to the principles of the Maillard reaction, it was hypothesized that the observed phenomenon might be attributed to the interaction between exogenous xylose and regenerated threonine, leading to the reformation of Thr-ARP. In particular, the carbonyl moiety of the supplementary xylose interacted with the amino group of the reformed threonine. Subsequently, this intermediate underwent rapid dehydration to form a Schiff base. This compound underwent cyclization to yield the corresponding N-substituted aldehyde amine. The aldose then proceeded through an Amadori rearrangement, ultimately transforming into Thr-ARP (Ames, 1990). To validate this conjecture, a model system comprising ^13^C^5^-D-xylose/Thr-ARP was established utilizing the isotope labeling technique. High-resolution mass spectrometry was employed to ascertain whether ^13^C^5^-Thr-ARP would be produced in the model reaction system during the heating process. The results obtained clearly demonstrated that the exogenous ^13^C^5^-D-xylose underwent cleavage and regeneration with Thr-ARP, leading to the formation of the Amadori compound. As illustrated in Fig. 4, the ion fragmentation mechanism revealed the generation of ^13^C^5^-Thr-ARP.

**Fig. 5:**
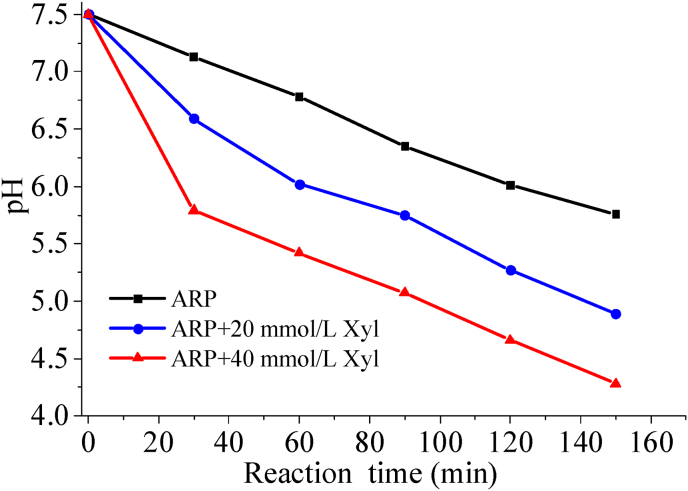
Time course of pH changes in the Thr-ARP model and Thr-ARP/Xyl model solutions during the thermal reaction.

It was important to highlight that pH played a critical role in the formation of pyrazine compounds (Martins and Van Boekel, 2005). An increase in pH could enhance the nucleophilicity of amino acids (Yu et al., 2012). In this study, elevated pH levels facilitated the Strecker degradation of regenerated threonine in the presence of GO, MGO, and other compounds, ultimately leading to the formation of pyrazine compounds. Conversely, a lower pH was not favorable for the formation of pyrazine compounds. During the thermal degradation process of Thr-ARP, as shown in Fig. 5, the pH gradually decreased; furthermore, an increase in the addition of exogenous xylose corresponded to a further reduction in pH. The drop in sample acidity likely resulted from organic acids, including acetic and glyceric acid, produced by 1-DX and 3-DX. This occurred through reaction pathways including hydrolytic α-cleavage and β-cleavage (Hofmann and Schieberle, 2000). The addition of xylose correlated positively with the production of deoxyxylosones through its cleavage, as illustrated in Fig. 1. This observation indicated that an increase in xylose concentration correlated with a more pronounced decrease in pH, which subsequently enhanced the inhibitory effect on the nucleophilicity of amino acids. The phenomenon was less favorable for the regeneration of threonine via Strecker degradation, ultimately affecting the formation of pyrazine compounds. Consequently, adding external xylose accelerated acidification during Thr-ARP solution heating, which in turn suppressed pyrazine production.

**Fig. 6:**
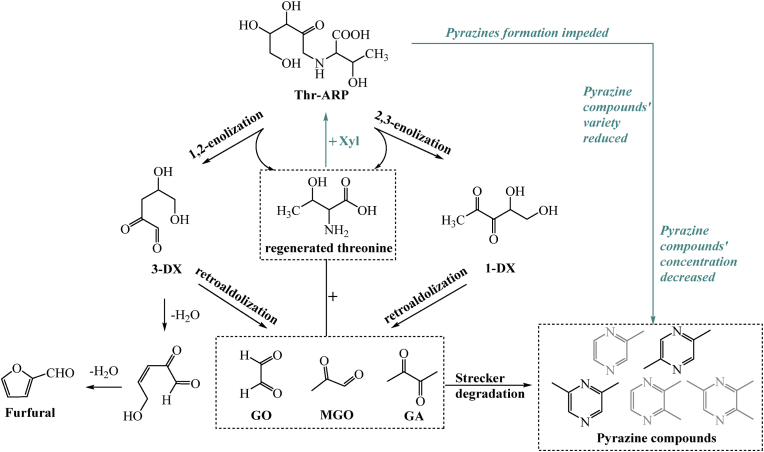
The proposed main pathways of exogenous xylose inhibiting Thr-ARP’ thermal reaction to generate pyrazine compounds.

Based on the analysis presented above, as illustrated in Fig. 6, the reaction pathway for exogenous xylose intervention in Thr-ARP degradation has been summarized. Compared to samples without the addition of exogenous xylose, the added xylose could engage in Maillard reaction initiation with threonine released from Thr-ARP thermal breakdown, ultimately yielding Thr-ARP via Amadori rearrangement (Cui et al., 2017). In contrast, in samples devoid of exogenous xylose, regenerated threonine did not participate in this reaction step. Instead, it could directly undergo Strecker degradation reactions with GO, MGO, and other substances produced by the retro-aldol reaction cracking of deoxyxylosones. This series of reactions culminated in the generation of pyrazines.

## 4 Conclusion

Isotope labeling was adopted to reveal the mechanism by which exogenous xylose inhibitied the thermal reaction of Amadori compounds to form pyrazine compounds. The research findings showed that exogenous xylose, competitively with glyoxal GO and MGO among others, reacted with regenerated threonine. Some of the regenerated threonine would be captured and rearranged by xylose, leading to the reformation of Thr-ARP. The process also contributed to a delay in the release of regenerated threonine. This resulted in a reduction in the concentration of regenerated threonine, which could undergo Strecker degradation with GO and MGO to produce pyrazine compounds. Consequently, the likelihood of effective collisions diminished. On the other hand, exogenous xylose was also subject to thermal degradation, resulting in the formation of 3-DX and other byproducts (Coleman and Chung, 2002). The breakdown of the deoxyxylosones produced organic acids, consequently lowering the solution's acidity (Haase et al., 2017). This reduction in pH would inhibit the nucleophilicity of regenerated threonine and diminish the reactivity with GO and MGO, consequently hindering the formation of pyrazine compounds. To summarize, the introduction of exogenous xylose and regenerated threonine facilitated the regeneration of ARP and induced a rapid decline in solution pH. The synergistic interaction between these two components resulted in a reduction in the concentration of pyrazine compounds produced through the thermal degradation of Thr-ARP, as well as a decrease in its overall richness.

## Declaration of competing interest

The authors declare that they have no known competing financial interests or personal relationships that could have appeared to influence the work reported in this paper.
