# Supplementary material for: Exogenous xylose trapped threonine to regenerate Amadori compounds and accelerated pH decline impeding pyrazines formation upon thermal treatment of Amadori compounds revealed by sotope labeling
Source: Curr Res Food Sci. 2026 May 6;12:101433. doi: 10.1016/j.crfs.2026.101433 (PMC13186052; doi:10.1016/j.crfs.2026.101433)
Supplement: Multimedia component 1 [file mmc1.docx]

**Exogenous xylose trapped threonine to regenerate Amadori compounds and accelerated pH decline impeding pyrazines formation upon thermal treatment of Amadori compounds revealed by Isotope labeling**

Pusen Chen ^†^, Baishun Hu ^§^, Zuman Dou ^⊥^, Fei Meng ^†,^ *, Qiong Deng ^§,^ *

^†^ Department of Food and Environmental Engineering, Chuzhou Vocational and Technical College, Chuzhou, 239000, PR China

^§^ Enshi Tujia and Miao Autonomous Prefecture Academy of Agricultural Sciences, Enshi 445000, China

^⊥^ College of Ocean Food and Biological Engineering, Jimei University, Xiamen 361021, China

**Author Information**

*Corresponding author: Fei Meng, Professor & Qiong Deng

(1) Fei Meng, Professor

Postal address*:* Department of Food and Environmental Engineering, Chuzhou Vocational and Technical College, Chuzhou, 239000, PR China.

(2) Qiong Deng, Professor

Postal address*:*Enshi Tujia and Miao Autonomous Prefecture Academy of Agricultural Sciences, Enshi 445000, China

**Figure captions**

**Figrue S1**. Concentration of furfural formed by thermal reaction of Thr-ARP with different concentrations of D-xylose (The initial concentration of Thr-ARP was 20 mmol/L, the reaction temperature was 120℃, and the initial pH was 7.5)

**Figrue S1**.
